# Maternal Vaccination for the Prevention of Infantile RSV Disease: An Overview of the Authorized, In-Progress, and Rejected Vaccine Candidates

**DOI:** 10.3390/vaccines12090980

**Published:** 2024-08-28

**Authors:** Georgios Papazisis, Xanthippi Topalidou

**Affiliations:** Clinical Research Unit, Special Unit for Biomedical Research and Education & Department of Clinical Pharmacology, School of Medicine, Aristotle University of Thessaloniki, 54124 Thessaloniki, Greece; xanthippitopalidou@gmail.com

**Keywords:** infant immunity, maternal antibody transfer, respiratory syncytial virus, maternal immunization, RSV vaccine, clinical trial, safety, immunogenicity

## Abstract

Respiratory Syncytial Virus (RSV) continues to pose a significant challenge, contributing to elevated hospitalization rates among children up to 5 years old, with a disproportionate burden on newborns and infants under 6 months old. The unique characteristics of the young immune system make it prone to altered responses to infections and vaccinations, requiring a tailored approach to disease prevention. The recent approval of the maternal RSV vaccine (brand name ABRYSVO) represents a pivotal advancement in preventive strategies among newborns and infants, marking a milestone in RSV research as the first market-approved maternal vaccine. The present review examines clinical trial data on both recent and previous vaccine candidates, as well as the licensed vaccine, focusing on the prevention of RSV disease in newborns and young infants through the passive acquisition of antibodies following maternal immunization. Additionally, it evaluates the safety profile of these vaccines.

## 1. Introduction

Infections exhibit a notably higher prevalence within the neonatal population compared to other age demographics. Based on data from the World Health Organization (WHO), it is reported that more than 550,000 infections per year result in fatal outcomes affecting primarily the low and middle-income countries (LMICs) [1]. Respiratory syncytial virus (RSV) substantially impacts morbidity and mortality, especially in early childhood. Following a brief period of decline in reported cases during the COVID-19 pandemic, there was a notable increase in the incidence of severe RSV disease [2]. According to the CDC, the seasonality pattern has altered with a tendency towards the spring and summer months after the pandemic, estimating the hospitalized patients until 5 years of age in the United States (U.S.) at a range number of 58,000–80,000 yearly [3]. According to a multicenter analysis including data extracted from 6 European countries, the annual hospitalization rate for RSV-associated cases exceeds 4% for neonates and infants up to 2 months old, whereas it ranges from approximately 0.1% to 1% for children aged 1 to 2 years old [4]. Interestingly, approximately half of hospital admissions and deaths in the hospital environment due to RSV infection of the lower respiratory tract affect newborns and infants up to 6 months of age [5]. In neonates, RSV infection can result in severe disease, necessitating mechanical ventilation hospitalization in the Neonatal Intensive Care Unit (NICU) [6].

Immunity in young children exhibits unique specificities compared to adult immune responses, necessitating a targeted approach to disease prevention in this sensitive age group. Immediate postpartum active immunization significantly protects newborns from serious diseases, including the Hepatitis B vaccine and the Bacillus Calmette-Guerine (BCG) vaccine [7]. However, the immunity induction requires time and possibly several doses of the vaccine to reach the desired immunity response [7]. Another strategy of providing protection to this age group is maternal immunization during pregnancy [7]. This approach offers a dual benefit, as both the mother and the newborn can acquire immunity against the disease, facilitating birth conditions and the perinatal period [7]. IgG antibodies originating from maternal immunization during pregnancy, pass transplacentally to the fetus, conferring efficient protection [8]. Antibodies provided by the mother are detectable for up to 6–12 months [9]. Concomitantly, evidence suggests that the presence of maternally acquired antibodies may diminish the B cell-induced immunity of the newborn through interventions at various stages of the immune response [9]. The CDC recommends protective vaccination against pertussis (Tdap vaccine), influenza, and COVID-19 in the period of pregnancy [10]. Recently, prevention of RSV has become available in this vulnerable age group, following the market authorization of a maternal RSV vaccine. ABRYSVO is the only market authorized vaccine from both the FDA and the EMA for maternal administration during pregnancy targeting the protection of newborns and infants [11,12]. 

The F protein of RSV is an attractive target for vaccine research due to its ability to induce sufficient host immunity and its stability among viral serotypes [13]. The G protein is not a popular option because of the instability among different RSV serotypes. However, the RSV G protein is being evaluated as a candidate for an adult RSV vaccine since a central domain tends to be invariable and capable of producing neutralizing activity [13]. Given the unclear function of the SH protein, it has been chosen as a novel antigen for an adult vaccine candidate with positive results. While this antigen does not seem to provide SH-specific neutralizing immunity, it limits viral replication and could be considered in future vaccine development, potentially as an adjuvant to another antigen [14,15]. The N protein could play an important role in RSV vaccination, participating actively in cytotoxic activity against the virus being developed as a vaccine candidate for adults [16]. Additionally, multiple modifications are being evaluated for the attenuation of the virus in the context of live attenuated vaccines for children. To our knowledge, maternal RSV vaccine candidates target only the F protein. 

This review article focuses on the analysis of the maternal vaccination in the context of RSV vaccines. A comprehensive review of the clinical trials of the currently approved and actively developed vaccines as well as the vaccine failures is provided. The analysis closely examines the outcomes related to preventing RSV disease in newborns and young infants, with a particular emphasis on the passive acquisition of antibodies following maternal immunization. Furthermore, it assesses the safety profile of these vaccines.

## 2. Materials and Methods

The search strategy involved extensive research of databases, including the Cochrane Database, MEDLINE, and EMBASE, as well as clinical trial registries, to encompass all previous and current vaccines developed as maternal RSV vaccine candidates. The search process was conducted using the following keywords and their synonyms: respiratory syndrome virus, maternal, and vaccine. Detailed information on the search algorithm is provided in the Supplementary Materials. 

Only active RSV maternal immunization agents were considered for inclusion in the review, without any limitations on publication date or language. All clinical studies that evaluated a vaccine candidate intended to immunize pregnant women and protect their infants, irrespective of their phase of development or conduct status, were included. Specifically, early-phase clinical trials involving non-pregnant women were included if the company had designed the vaccine candidate as a maternal vaccine. Excluded from the research were non-original studies, reviews, meta-analyses, and preclinical studies. Additionally, clinical trials of passive immunization agents for infant immunization were excluded. The data was last updated on 2 May 2024. A narrative synthesis approach was employed to analyze the gathered data. A modified version of the PRISMA flow diagram in the Supplementary Materials depicts the data screening process.

## 3. Results

Maternal vaccination for the prevention of infantile RSV disease is recently a key focus of research, given the availability of an approved vaccine. A comprehensive overview of current and previous clinical trials is presented in Table 1.

### 3.1. Abrysvo

Pfizer Inc. developed Abrysvo to induce specific immunity against the prefusion form of the F RSV structural protein. In August 2023, regulatory authorities, beginning with the FDA and followed by the EMA, provided a positive opinion for its marketing authorization as a maternal vaccine. The recommended vaccination window during pregnancy extends from 32 to 36 weeks of gestation to achieve protection for the newborn and infant up to 6 months of age against RSV-associated lower respiratory tract disease (LRTD) and severe manifestations of LRTD [21].

A phase I/II trial (NCT03529773) included healthy nonpregnant women among other participants and tested different dose levels, adjuvant use, and a co-administration with the seasonal inactivated influenza vaccine (SIIV) in phase II. Notably, a greater increase in the geometric mean titers (GMTs) of neutralizing antibodies was observed in the female participants, particularly those with low antibody levels at baseline. Although a decrease was noted within a year, levels remained 4–5 times higher compared to baseline, supporting the principle of maternal vaccination in the late second or third trimester for optimal peripartum protection. The high IgG1 titers observed, which cross the placenta, are also promising [17]. Additionally, the administration of both RSV and influenza vaccines did not result in significant changes in specified RSV immune responses. However, concerning influenza immunity, young adults in the study exhibited reduced responsiveness to the SIIV following coadministration [18]. A sub-study conducted one year after the initial vaccination included revaccination of some participants from the parent study. This sub-study demonstrated successful induction of immunogenicity following revaccination [36].

In the subsequent phase IIb study (NCT04071158), healthy nonpregnant women were recruited for a non-inferiority trial evaluating the coadministration of RSV and tetanus, diphtheria, and acellular pertussis (Tdap) vaccines. According to the study results, the non-inferiority criteria for antibody-induced immunity were met for the desired pathogens, except for immunity against pertussis. Coadministration did not yield similar immunogenicity results as the single Tdap administration for pertussis. These data further support the concept of maternal vaccination against multiple pathogens [19]. Pregnant women between the 24th and 36th gestational weeks were vaccinated in another phase IIb trial (NCT04032093) with two different dose levels and formulations, one with adjuvant and one without. An interim analysis of the 6-month data provided evidence supporting the activation of immunity during pregnancy, eliciting maternal neutralizing antibody production and transmission to the infant. Post hoc analysis indicated that the acquired antibodies prevented infantile disease at high rates. Measurements in infants revealed higher antibody levels with the unadjuvanted formulation, which were not correlated with the gestational age spectrum during vaccination. Pregnancy, labor, and delivery-related adverse events, including preterm deliveries, exhibited a similar distribution between the placebo and vaccine groups. A rate of 3.7% of newborns were premature across all groups. Investigators noted that most congenital anomalies were not attributed to the vaccination and were of mild severity [20]. Based on updated information provided in the vaccine’s package insert, preterm births were found to be unevenly distributed between the active and placebo groups, with rates of 5.3% and 2.6%, respectively [21]. A phase 2a RSV challenge study (NCT04785612) conducted in young adults, including non-pregnant female participants, demonstrated effective protection against symptomatic manifestation of RSV infection following RSV challenge [22].

The MATISSE trial (NCT04424316), a phase III trial with a large number of participants, enrolled pregnant women in low-risk pregnancies between 24 and 36 weeks of gestation. According to interim analysis, the vaccine effectively prevented RSV medically attended severe lower respiratory tract illness within the first 3 months after birth in infants at a rate of 81.8%. However, the corresponding efficacy rate for lower respiratory tract illness was 57.1%, failing to meet the statistical success criterion. The protection rate for severe disease within the first 6 months after birth exceeded 69%. The vaccine was proven safe for both pregnant women and their infants, while final data analysis is anticipated. Specifically, regarding the safety profile, preeclampsia occurred in 1.8% of the vaccine recipients compared to 1.4% among placebo recipients, with comparable rates for fetal distress syndrome. Premature delivery experienced 0.8% of the vaccine recipients and 0.6% of the placebo recipients, while stillbirth and spontaneous abortion were reported at even lower rates. Serious adverse events regarding pregnancy outcomes occurred in a comparable distribution between the groups [23]. As summarized in the vaccine’s package insert, the rate of recorded preterm births was 5.7% for the Abrysvo recipients and 4.7% for the participants in the control group [21]. Another phase III trial (NCT05096208), which also included female participants, proved the consistent safety and reactogenicity of three different lots of the vaccine [24]. The MORISOT trial (NCT06325657), a recently announced phase III clinical trial, intends to investigate the effects of vaccinating pregnant women living with HIV and their infants. Eligible participants are female individuals in their 24th to 36th week of pregnancy, demonstrating stable HIV disease status.

In the context of administering safe drugs, the continuous reevaluation of post-marketing data remains vital, placing pharmacovigilance at the center of interest for researchers. Additional studies should assess whether a causal relationship exists between the reported adverse events and the vaccination, and they should also detect any further significant adverse events that have not yet been reported. Regarding preterm births, pregnancies of high risk should also be included in the clinical trials. Careful monitoring of the safety profile, with a specialized report of the already existing concerns, is mandated by the FDA through surveillance systems such as the Vaccine Adverse Event Reporting System (VAERS).

### 3.2. RSV MAT

The maternal vaccine candidate developed by GSK plc, targeting the same protein as Abrysvo, was deemed safe in the first-in-human clinical trial (NCT03674177) conducted in non-pregnant women. Immunization data from this phase I trial supported the further evaluation of the vaccine during pregnancy [25].

In the phase II clinical trial (NCT04126213), the vaccine was evaluated during 28 to 33 weeks of pregnancy. An acceptable safety profile was demonstrated, with similar rates of adverse events observed between vaccine recipients and placebo recipients. Pregnancy and peripartum abnormalities, including preterm labor, preterm birth, or congenital anomalies, were observed to have a similar trend between the groups. While an increased reporting rate of hypertension and preeclampsia was noted in the active immunization group, it did not exceed the rates observed in the general pregnant population. Specific antibody titers were measured at elevated levels in both vaccinated mothers and their infants [26]. In a co-administration phase II study (NCT04138056), the safety and immunogenicity profile of Diphtheria-Tetanus-Pertussis co-vaccination with the RSV vaccine candidate were examined in non-pregnant women. The study found no evidence of interference between the vaccinations, and specific RSV antibody reactions remained evident up to one to one and a half years postvaccination. However, interference was observed with the Diphtheria-Tetanus-Pertussis vaccination, with no clinical significancy for the diphtheria and tetanus antigens and unknown clinical interpretation for the pertussis component of the vaccination [27].

The GRACE trial, a phase III clinical study (NCT04605159), was designed to evaluate the safety profile and efficacy rates of vaccination in pregnant individuals and their infants. The vaccine efficacy in protecting infants against medically assessed RSV-lower respiratory tract disease was found to be satisfactorily high. However, significant safety signals emerged, leading to the early discontinuation of the trial before enrolling the planned number of participants. Specifically, an association with increased possibilities of preterm birth was observed, with 6.8% of infants born to vaccinated mothers being preterm compared to 4.9% in the placebo group [33]. Consequently, another phase III clinical study (NCT04980391) in high-risk pregnancies was prematurely discontinued, and a re-vaccination trial (NCT05229068) involving previously vaccinated individuals from earlier trials was withdrawn. Additionally, the phase III (NCT05169905) clinical trial in non-pregnant girls and females included only nine participants due to the decision to terminate the study. Concurrently, another already ongoing phase III trial (NCT05045144) enrolled non-pregnant women for testing different lots and evaluating co-administration with the influenza vaccine in this population. Recently, the company initiated a safety-focused phase IIIb clinical trial (NCT05705440) without any intervention. This open-label trial involves the follow-up of participants from all previous trials to monitor safety parameters among participants from both the active and placebo groups.

Previously, GSK had developed another vaccine candidate (GSK3003891A) based on the same protein. However, the formulation was found to be unstable, and this particular vaccination was not further pursued.

### 3.3. ResVax

A nanoparticle vaccine encoding the F RSV protein was developed by Novavax, Inc. The vaccine candidate underwent testing in a phase I clinical trial (NCT01290419) in 2011, involving young male and female participants. The results from this trial supported further evaluation in phase 2 trials, as the vaccine demonstrated no association with toxicity and elicited an increase in specific antibody titers [28]. In the subsequent phase II studies (NCT01704365, NCT01960686), healthy women aged 18 to 35 years were enrolled to investigate the dose range and adjuvant addition. The trials yielded no safety concerns, and cases of RSV were less common after vaccination. Based on the results, the high antigen dose combined with the middle adjuvant dose was selected for further evaluation as a maternal vaccine [29,30]. Another phase II clinical trial (NCT02247726) enrolled pregnant women in the third trimester with a follow-up period of one year for their infants. Vaccination provided protection against severe RSV disease for both the maternal participants and their infants, with no significant safety issues. Infants exhibited high levels of antibodies measured from cord blood, with an average half-life of 40 days [31]. In the phase III clinical trial (NCT02624947) of the vaccine, a larger number of pregnant women between 28 and 36 weeks of gestation age participated. No serious safety signals were observed, with similarity noted between the groups in terms of low birth weight, small for dates, premature birth, and intrauterine growth restriction. However, vaccine efficacy did not meet the prespecified criterion for RSV-specific medically-significant lower respiratory tract infection. Up to 3 months of age, the rate of vaccine efficacy for this primary outcome was 39.4%, while it increased to 58.8% for disease manifesting with severe hypoxemia in the same period. Notably, similar rates of vaccine efficacy were achieved for all-cause lower respiratory tract infections, including related hospitalizations and severe hypoxemia, which is an important finding. A higher incidence of protection against hospitalizations and severe cases in LMICs emerged from the data of this study. This noteworthy observation highlights the need for further investigation in subsequent vaccine studies since the trial lacked the necessary statistical power to assess efficacy on a country-by-country basis [34]. The vaccine is no longer part of the company’s pipeline [37].

### 3.4. mRNA-1345

Utilizing mRNA vaccine technology and lipid nanoparticles as a transport system, Moderna Inc. manufactured mRNA-1345 targeting the RSV F protein. In the phase I clinical trial (NCT04528719), the vaccine was administered to healthy young adults, including women, with promising outcomes for further development of the vaccine as a maternal candidate. Elevated levels of antibody titers were observed within a 6-month period after vaccination [32]. A subsequent phase II clinical trial (NCT06143046) is currently enrolling pregnant women between 28 and 36 weeks of gestation, with the intention of also following up on their infants.

### 3.5. V-306

The V-306 vaccine candidate, developed by Virometix AG, has a particular vaccine target of an antigen epitope (FsIIm) of the RSV F protein. In the phase I clinical trial (NCT04519073), the vaccine candidate did not demonstrate the expected induction of neutralizing and IgG specific immunity. A possible development of V-306 presupposes vaccine formulation alterations [35].

## 4. Discussion

The recent licensure of maternal vaccines heralds a significant stride in the prevention of RSV among the vulnerable demographic of newborns and young infants. The United States Food and Drug Administration (FDA) set a narrower window between 32 and 36 weeks of gestation as a maternal vaccination indication, while the European Medicines Agency (EMA) advocates for vaccination within the broader range of 24 and 36 weeks of gestation [12,38]. Preterm infants, particularly those born prematurely, are disproportionally affected by RSV-related illness, experiencing notably higher rates of hospitalization. These findings emphasize the need for potential immunization early during pregnancy [39]. The FDA mandated a specialized report on preterm births and hypertension during pregnancy using real-world data through the VAERS and established precise pharmacovigilance objectives for ABRYSVO. Detailed post-marketing pharmacovigilance trials are delineated by the FDA in order to ensure safety [40]. It is essential to highlight that, despite the lack of data concerning the frequency and complications of RSV infection during pregnancy, emerging evidence suggests a plausible association between RSV infection and preterm delivery [41]. Similarly, available data regarding influenza indicate a correlation with abortions, preterm births, and stillbirths [42]. Consequently, the systematic collection of data regarding peripartum outcomes subsequent to RSV infection during pregnancy holds the potential to modify vaccination strategies.

Reporting of past failures in vaccine development can provide crucial information for optimizing developmental techniques and offer evidence of possible adverse events that researchers should consider for future vaccines. The RSV MAT vaccine raised significant safety concerns due to its association with a statistically significant increase in preterm births, which ultimately led to its discontinuation during phase III of development [33]. A safety-focused clinical trial is currently ongoing to explore safety parameters from previous clinical trials and investigate their association with preterm births. In the case of the ResVax, the primary outcome was not achieved; however, it was shown that infants born to vaccinated mothers were less likely to develop all-cause pneumonia. The researchers emphasized that future vaccine efforts should address LRTI with severe hypoxemia and conduct studies stratified by country or national economic status [34]. V-306 did not result in an increase in neutralizing antibody titers. Nevertheless, it was demonstrated that the technique of using a synthetic virus-like particle-based vaccine is an effective method that could be further explored with a different antigenic site of the F protein or other RSV proteins as a suggestion for further vaccine development [35].

Recently, Nirsevimab, which is a long-acting monoclonal antibody, was market-approved as an RSV passive immunization agent for infants. Immunizing infants < 8 months within their first RSV season and infants and children 8–19 months of age with high-risk factors for severe infection belong to the indications of Nirsevimab [43]. To date, there is no evidence to indicate that either of the two agents is more effective. However, simultaneous administration of both immune agents is not substantiated, except in specific, rare cases where it is considered clinically useful [44]. Conducting studies to assess the effects of each medication under specific circumstances could facilitate the optimization of population benefits through an individualized clinical approach. A recent analysis funded by the National Institute for Health Research suggested that infants benefit from the use of either long-acting monoclonal antibodies or maternal vaccination. An additional protective effect is also expected for pregnant individuals in the case of the maternal vaccination [45]. Averting RSV cases in vaccinated mothers could reduce the overall burden of the disease. Modified immune responses occur during pregnancy, increasing the mother’s susceptibility to severe manifestations of infections [46]. Fetal acquisition of maternal antibodies initiates around the 13th week of gestation and escalates after the 28th week, intending to protect the infant during the critical phase of immune system adaptation [47]. Additionally, the maternal vaccine is not as expensive as the monoclonal antibody, making it an attractive option for establishing cost-effective vaccine strategies, especially in LMICs. Based on the “maternal-driven immune education” hypothesis, it is believed that early exposure to antibodies through the mother could prime the infant’s immunity induction, potentially resulting in long-lasting effects [48].

Prioritizing widespread access to maternal vaccination in LMICs is a high priority. Gavi, the Vaccine Alliance, advocated RSV maternal vaccine efforts through its Vaccine Investment Strategy. Infants uninfected but exposed to HIV may experience higher rates of morbidity because of infectious diseases such as RSV, making maternal immunization important for the prevention of infectious diseases in this population [49]. A specific mathematical model predicted a prevention rate of RSV-related deaths of 94% for vaccination at 24 weeks of gestation age and 82% for vaccination at 32 weeks of gestation age with data extracted from trials in LMICs. Considering that the out-of-hospital mortality rates are three times higher than in-hospital rates, over 3000 deaths in infants under 6 months of age could be prevented through maternal vaccination [50]. Tailored models projected significant potential for an RSV maternal vaccine to mitigate disease impact in infants within 6 months of life across 73 Gavi-supported countries. Such analyses serve as crucial resources for guiding targeted prevention strategies of global organizations such as Gavi and the World Health Organization (WHO) [51]. Mathematical models provide valuable insights into the cost-effectiveness of vaccination strategies in these settings. Results from a mathematical model concerning RSV-associated mortality within the first 6 months of life indicate that vaccination could substantially have an impact in reducing the death rates in LMICs [52]. An analysis based on data from Kenya and South Africa illustrates that, considering the disease prevalence in these countries among infants younger than 6 months, preventive methods would offer cost-effective solutions [53]. Further cost-effectiveness analyses conducted across various geographical distributions, including LMICs, and tailored to the epidemiological characteristics of the virus can actively participate in the global implementation of RSV maternal vaccination. Critical factors such as vaccine manufacturing, delivery, affordability, and financing need to be carefully assessed and determined.

## 5. Conclusions

Summarizing the results of maternal RSV vaccination provides an overview of its development over the years. The report of vaccine failures highlights the significant points that must be taken into consideration for future vaccine development and pharmacovigilance. In-depth research and analysis of the reasons for vaccine withdrawal or failure may unveil potential mediating mechanisms. Real-world clinical data are essential to address gaps in safety and efficacy of the already-approved vaccine, while cost-effectiveness analyses can inform the guidelines on RSV prevention. Long-term follow-up studies and rigorous safety monitoring, by encouraging the report of any adverse events, as well as clinical data on specific populations, such as immunosuppressed pregnant women, are needed for the further vaccine evaluation.

## Figures and Tables

**Table 1:** Clinical trials of the maternal RSV vaccines categorized by clinical study status.

Vaccine	Registration Number	Phase	Population	Results
Completed clinical trials				
Abrysvo (Pfizer)	NCT03529773	I/II (2018–2020)	adults including non-pregnant female participants	- Higher titers of neutralizing antibodies in female participants, supporting the principal of maternal vaccination, levels above baseline within a year - no differences in RSV responses after coadministration with influenza vaccine (reduces responsiveness to influenza vaccination) [17,18]
	NCT04071158	IIb (2019)	non-pregnant female participants	- non-inferiority coadministration trial with tetanus, diphtheria and acellular pertussis vaccine (Tdap) - non-inferiority of coadministration was proved for all the pathogens except for pertussis, supporting vaccination against multiple pathogens [19]
	NCT04032093	IIb (2019–2021)	pregnant female participants (24th–36th gestational week)	- interim analysis: maternal neutralizing antibody production and adequate infant transmission - post-hoc analysis: adequate prevention of infantile disease [20] - 5.3% of preterm births in the active group compared to 2.6% in the placebo group [21]
	NCT04785612	IIa (2020–2021)	young adults including non-pregnant female participants	- RSV challenge study - effective against symptomatic disease after RSV exposure [22]
	MATISSE NCT04424316	III (2020–2023)	pregnant female participants (24th–36th gestational week), low-risk pregnancy	- interim analysis - avoidance of RSV-severe lower respiratory tract disease in the first 3 months of life in 81.8% - efficacy rate for lower respiratory tract illness was not statistically significant (57.1%) - protection rate against severe disease within the first 6 months after birth exceeded 69% [23] - premature births were recorded at a rate of 5.7% for the vaccine recipients and 4.7% for the placebo recipients [21]
	NCT05096208	III (2021–2022)	adults including female participants	- consistency and safety of three different vaccine lots [24]
RSV MAT (GSK)	NCT03674177	I (2018–2019)	non-pregnant female participants	- positive data for further evaluation of the vaccine during pregnancy [25]
	NCT04126213	II (2019–2021)	pregnant female participants (28th–33rd gestational week)	- pregnancy and peripartum abnormalities in similar rates between groups, more cases of hypertension and preeclampsia in the active group without exceeding the general pregnant population rates - elevated titers of antibodies for mothers and their infants [26]
	NCT04138056	II (2019–2021)	non-pregnant female participants	- no interference in RSV-related outcomes after coadministration with Tdap and altered responses for the Tdap pathogens with no (diphtheria and tetanus) or unknown (pertussis) clinical significancy [27]
	NCT05045144	III (2021–2022)	non-pregnant female participants	- different vaccine lots and co-administration with influenza vaccine
ResVax (Novavax)	NCT01290419	I (2010–2011)	young male and female participants	- positive results, no association with toxicity, increase of antibody titers - supported further evaluation [28]
	NCT01704365	II (2012–2013)	non-pregnant female participants	- no serious safety concerns were raised - specific antibody immunity was triggered [29]
	NCT01960686	II (2013–2014)	non-pregnant female participants	- the high antigen dose formulation achieved high titers of antibodies remaining until 3 months post-vaccination and proved safe [30]
	NCT02247726	II (2014–2016)	pregnant female participants (33rd–35th gestational week)	- protection against severe disease for both mothers and their infants, with high antibody titers for the infants - no significant safety issues [31]
Currently ongoing clinical trials				
mRNA-1345	NCT04528719	I (2020–2024)	young adults including female participants	- promising outcomes for further development as a maternal candidate with elevated antibody titers within a 6-month period [32]
	NCT06143046	II (2023–2026)	pregnant female participants (28th–36th gestational week)	-
ABRYSVO	MORISOTNCT06325657	III (2024–2026)	pregnant female participants living with HIV (24th–36th gestational week)	-
RSV MAT	NCT05705440	IIIb (2023–2025)	participants from all previous trials	- safety monitoring
Discontinued clinical trials				
RSV MAT	GRACE trialNCT04605159	III (2020–2023)	pregnant female participants (24th–34th gestational week)	- high protection rates against RSV lower respiratory tract disease - Safety signals: risk of preterm birth (6.8% in the active group and 4.9% in the placebo group) [33] - termination of the study
	NCT04980391	III (2021–2023)	pregnant female participants (24th–36th gestational week), high-risk pregnancy	- premature discontinuation based on the findings of the GRACE trial
	NCT05229068	III (2022)	re-vaccination trial	- withdrawn, based on the findings of the GRACE trial
	NCT05169905	III (2022)	non-pregnant female participants	- early termination of the trial
Trials that did not achieve the outcome				
ResVax	NCT02624947	III (2015)	pregnant female participants (28th–36th gestational age)	- similar rates of pregnancy and birth adverse events between groups with no safety signals - Vaccine efficacy did not meet the primary outcome for protection against RSV lower respiratory tract infection within 3 months of age (39.4%) - VE of 58.8% for disease manifesting with severe hypoxemia [34]
V-306 (Virometix)	NCT04519073	I (2020–2022)	non-pregnant female participants	- the expected induction of antibodies and immunity was not achieved [35]

## Supplementary Materials

The following supporting information can be downloaded at: https://www.mdpi.com/article/10.3390/vaccines12090980/s1, Search methodology; PRISMA flow diagram. Reference [54] is cited in the Supplementary Materials.
