# Supplementary material for: Maternal Vaccination for the Prevention of Infantile RSV Disease: An Overview of the Authorized, In-Progress, and Rejected Vaccine Candidates
Source: Vaccines (Basel). 2024 Aug 28;12(9):980. doi: 10.3390/vaccines12090980 (PMC11435746; doi:10.3390/vaccines12090980)
Supplement: Supplementary file 1 [file vaccines-12-00980-s001.zip › vaccines-3115737 - Supplementary Figures.pdf]

Modified PRISMA 2020 flow diagram

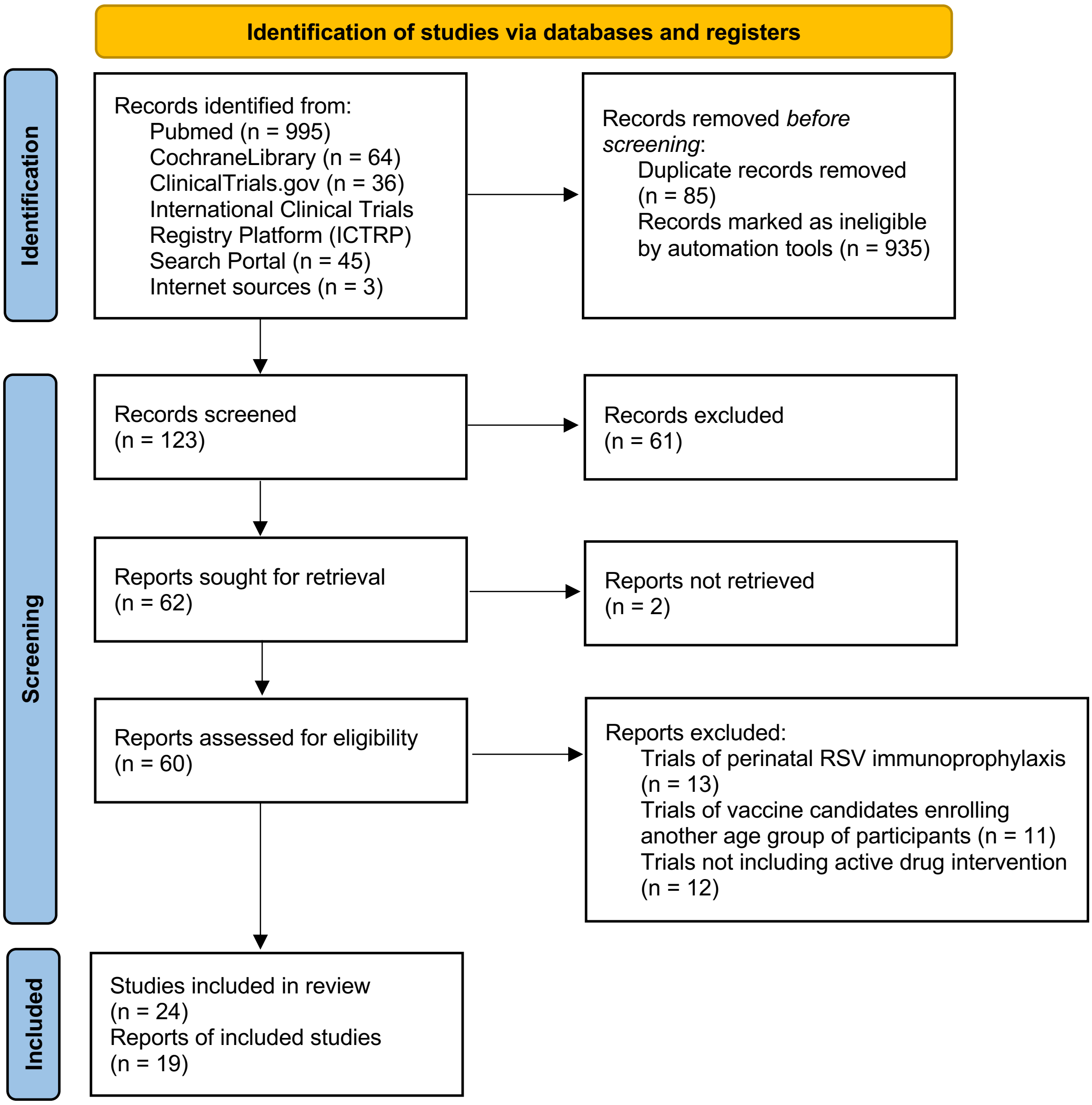

From: Page MJ, McKenzie JE, Bossuyt PM, Boutron I, Hoffmann TC, Mulrow CD, et al. The PRISMA 2020 statement: an updated guideline for reporting systematic reviews. BMJ 2021;372:n71. doi: 10.1136/bmj.n71

For more information, visit: http://www.prisma-statement.org/
